# Temporal trends of case-fatality in patients undergoing dual-injection coronary chronic total occlusion recanalization

**DOI:** 10.1007/s00392-023-02298-x

**Published:** 2023-09-11

**Authors:** Recha Blessing, Karsten Keller, Zisis Dimitriadis, Thomas Münzel, Tommaso Gori, Lukas Hobohm

**Affiliations:** 1 Department of Cardiology, Cardiology I, University Medical Center Mainz (Johannes Gutenberg-University Mainz), Langenbeckstrasse 1, 55131 Mainz, Germany; 2 Center for Thrombosis and Hemostasis (CTH), University Medical Center Mainz (Johannes Gutenberg-University Mainz), Mainz, Germany; 3 Medical Clinic VII, Department of Sports Medicine, University Hospital Heidelberg, Heidelberg, Germany; 4 Division of Cardiology, Department of Medicine III, University Hospital Frankfurt, Goethe University Frankfurt Am Main, Frankfurt, Germany; 5 German Center for Cardiovascular Research (DZHK), Partner Site Rhine Main, Berlin, Germany

**Keywords:** Chronic total occlusion (CTO), Percutaneous coronary intervention (PCI), Coronary artery disease (CAD), Recanalization

## Abstract

**Aims:**

Recently, interventional techniques and material to treat chronic total occlusion (CTO) with percutaneous coronary intervention (PCI) have evolved significantly. Nevertheless, it is still unknown whether this progress improved treatment success and patients’ outcome. In a nationwide sample, we sought to analyze trends of patients’ characteristics, complications and in-hospital case-fatality of patients undergoing CTO revascularization in Germany.

**Methods and Results:**

We analyzed data on characteristics, treatments, and in-hospital outcomes for all coronary artery disease (CAD) patients (ICD-code I25) undergoing dual-injection CTO recanalization (OPS procedural code: 8–839.9) in Germany from 2009 to 2020. Overall, 4,998,457 inpatients aged ≥ 18 years with diagnosis of CAD were treated in German hospitals in this period. Among these, 52,879 patients (1.1%) underwent CTO recanalization. Annual number of CTO PCIs increased from 1263 in 2009 to 6435 in 2020 (β 3.48 [95% CI 3.44–3.52]; *p* < 0.001) in parallel with a significant decrease of case-fatality (2.2% in 2009 to 1.4% in 2020; β  – 0.60 [95% CI  – 0.82 to  – 0.39]; *p* < 0.001). Overall, 754 (1.4%) patients with CTO recanalization died during the in-hospital stay and in-hospital case-fatality grew exponentially with age (β 0.82 [95% CI 0.73–0.90]; *p* < 0.001). Significant predictors of in-hospital case fatality with an OR > 3 were cancer, stroke, hemopericardium, acute renal failure, pulmonary embolism and shock.

**Conclusion:**

Annual number of CTO procedures performed in Germany increased from 2009 to 2020 with a concomitant anti-proportional decrease in the case-fatality. Our findings may help to draw more attention to predictors of in-hospital case fatality in patients hospitalized for CTO recanalization.

**Graphical abstract:**

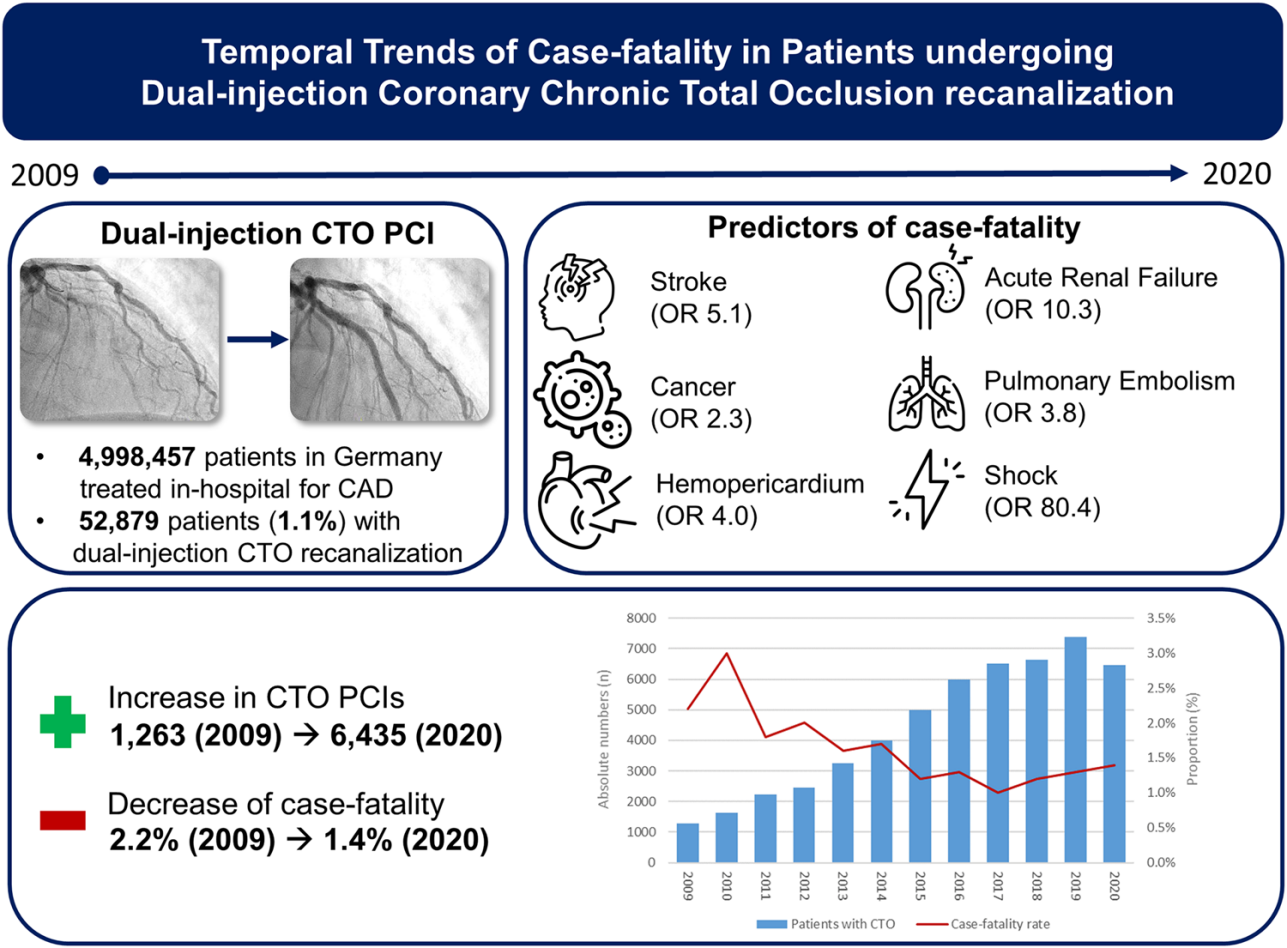

## Introduction

Chronic total occlusions (CTOs) of coronary arteries are diagnosed in 16–20% of the patients who undergo coronary angiography [1–3]. Despite the high and increasing incidence, treatment of CTO requires high specialization and advanced skills and is, therefore, not performed in every cardiac catheterization laboratory and not systematically by all interventional cardiologists [4, 5]. The high incidence of complications and the lack of clear clinical and prognostic benefits limit the penetration of this type of procedure. However, clinical studies show improved health status and quality of life following successful CTO recanalization [1, 6–8]. In contrast, it remains controversial whether successful CTO PCI may impact survival [7, 9–13]. Although CTO PCI remains a complex procedure for the interventional cardiologist, introducing new materials, evolved procedural techniques and modified hybrid treatment algorithms increased the technical success to almost 90% with an acceptable complication rate in experienced CTO intervention centres [14]. Thus, more patients with complex coronary anatomy or a high cardiovascular risk profile as diabetes mellitus undergo successful CTO recanalization for complete revascularization and reduction of the ischemic zone [15–21]. Data from large cohorts that investigate the trends of patients’ characteristics, success rate, complications, and in-hospital case-fatality are limited. Thus we aimed to close this gap, investigating data of the very large German nation-wide inpatient sample including all patients, who underwent CTO recanalization in Germany between the years 2009 and 2020.

## Material and methods

### Data source

Statistical analyses were performed on our behalf by the Research Data Center (RDC) of the Federal Bureau of Statistics (Wiesbaden, Germany). Aggregated statistics were provided from RDC based on a predefined SPSS code (IBM Corp. Released 2011. IBM SPSS Statistics for Windows, Version 20.0. IBM Corp: Armonk, NY, USA), which we had previously generated and supplied to the RDC (source: RDC of the Federal Statistical Office and the Statistical Offices of the federal states, DRG Statistics 2020, own calculations).

### Aim of the study, diagnoses, procedural codes, and definitions

We report on the characteristics and temporal trends of patients undergoing dual-injection CTO recanalization (OPS-code 8-839.9) in Germany between Jan 1st of 2009 until 31st December of 2020 and identify independent predictors of in-hospital case fatality as a primary outcome. As presented below, data regarding coexisting conditions and complications were collected using the corresponding diagnostic and procedural codes (OPS and ICD-10-GM codes).

### Ethical aspects

Since our study did not comprise direct access by the investigators to individual patient data but only an access to summarized results provided by the RDC, approval by an ethics committee as well as patients’ informed consent were not required, in accordance with German law.

### Statistical analysis

Patients’ characteristics are presented as absolute and relative numbers (%) or median [interquartile range] and are analyzed using the Wilcoxon–Whitney *U* test for continuous variables and Fisher’s exact or chi^2^ test for categorical variables, as appropriate. Temporal trends regarding hospitalizations of patients with CTO recanalization, procedural approaches, and in-hospital mortality over time and with increasing age were estimated using linear regression analyses. The computed results are presented as beta (β)-estimates with corresponding 95% confidence intervals (CI). Logistic regression models were applied to investigate associations between patients’ characteristics and adverse in-hospital events including in-hospital death. Results were presented as Odds Ratios (OR) and 95% confidence intervals (CI). The multivariate regression models were adjusted for age, sex, cancer, heart failure, chronic obstructive pulmonary disease (COPD), essential arterial hypertension, renal insufficiency and diabetes mellitus. This approach for adjustment was selected to reach a widespread independence of the investigated factors and diseases on in-hospital case-fatality (independent of these adjusted drivers of in-hospital death as well as age and sex).

All statistical analyses were carried out with the use of SPSS software (IBM Corp. Released 2011. IBM SPSS Statistics for Windows, Version 20.0. IBM Corp: Armonk, NY, USA). Only *P*-values of < 0.05 (two-sided) were considered to be statistically significant. No adjustment for multiple testing was applied.

## Results

### Baseline characteristics and temporal trends of CTO recanalization in Germany

We analyzed data of 4,998,457 inpatients aged ≥ 18 years with diagnoses of CAD in Germany; among these, 52,879 (1.1%) had diagnosis of CTO recanalization. The majority of these patients were male (80.5%) with median age of 66 years and a median length of in-hospital stay of 3 days.

**Fig. 1:**
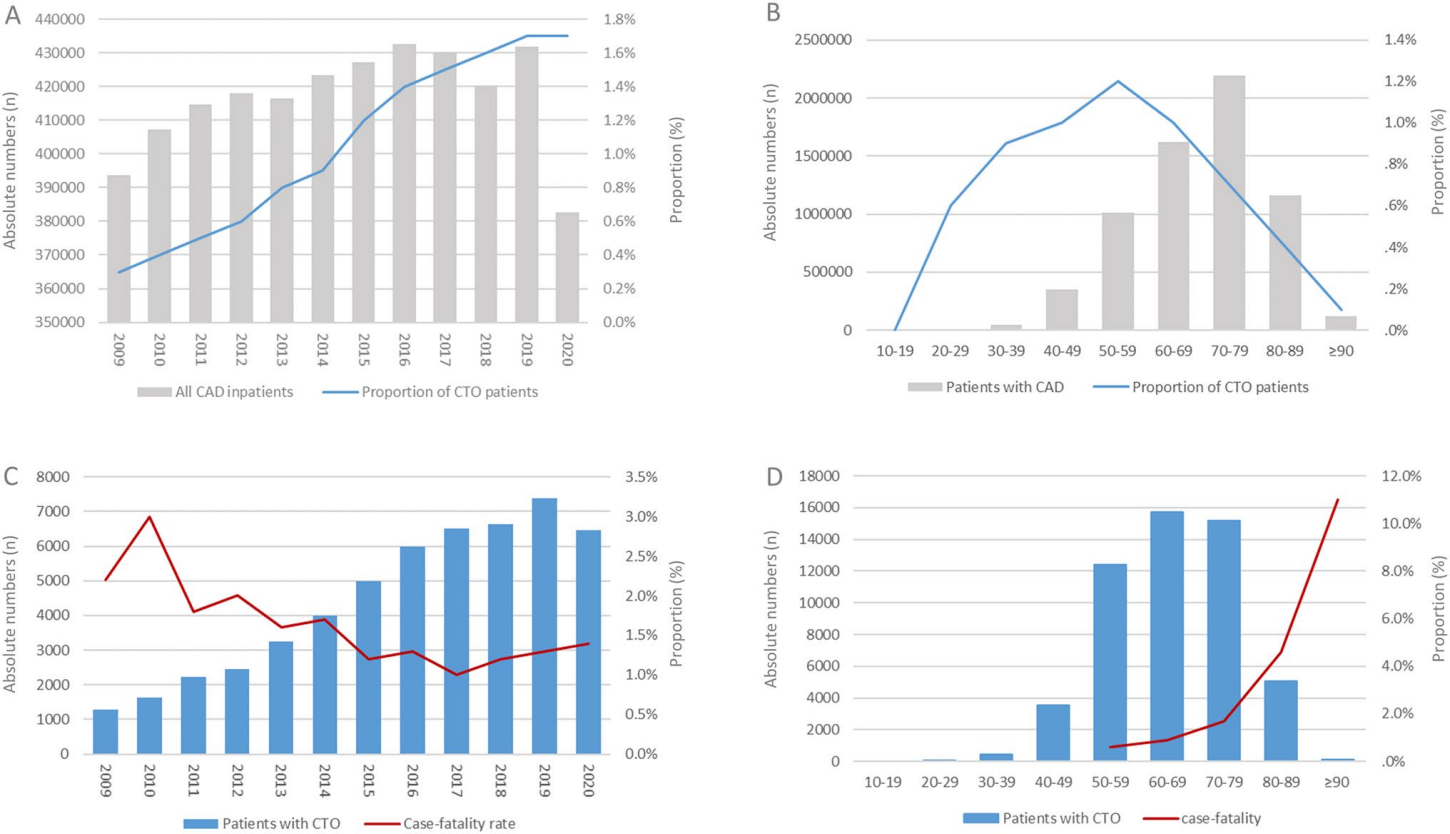
Proportion rate of CTO diagnosis in patients with CAD across years (**A**) and age-decades (**B**). Case-fatality rate in patients with CTO PCI across years (**C**) and age-decades (**D**) in Germany

The annual number of CTO PCIs increased significantly from 1263 (0.3%) in 2009 to 6435 (1.7%) in 2020 (β 3.48 [95% CI 3.44–3.52]; *p* < 0.001) in parallel with anti-proportional decrease of case-fatality (2.2% in 2009 to 1.4% in 2020; β  – 0.60 [95% CI  – 0.82 to  – 0.39]; *p* < 0.001) (Fig. 1A and C). While the proportion of CTO recanalization in relation to the total numbers of patients with CAD increased until the 6th decade, this proportion decreased with increasing age (β  – 0.37 [95% CI  – 0.38 to  – 0.36]; *p* < 0.001) (Fig. 1B). The case-fatality rate was highest in 8th and 9th age decade (Fig. 1D).

### Comparison of survivors and non-survivors

Non-survivors were older and had more frequent comorbidities such as cancer, heart failure, atrial fibrillation, COPD, chronic renal insufficiency, diabetes mellitus and peripheral artery disease (PAD) (Table 1). As expected, non-survivors were coded more frequently with myocardial infarction (536 [71.1%] vs. 8,035 [15.4%], *P* < 0.001). Non-survivors were more often observed with ischaemic stroke, pneumonia, acute renal failure, ventricular arrhythmia, deep vein thrombosis and pulmonary embolism (Table 1). Pericardial effusion (47 [6.2%] vs. 804 [1.5%], *P* < 0.001) and hemopericardium (17 [7.5%] vs. 211 [0.4%], *P* < 0.001) within CTO recanalization was observed considerably more often in non-survivors compared to survivors.

**Table 1:** Baseline characteristics, medical history and presentation of 52,879 patients undergoing CTO PCI (cumulative data of the years 2009–2020)

Parameters	All patients with CTO PCI (*n* = 52,879)	Non-survivors (*n* = 751; 1.4%)	Survivors (*n* = 52,125; 98.5%)	*P*-value
Age	66 (56–72)	76 (67–81)	66 (57–74)	** < 0.001**
In-hospital stay (days)	3 (2–5)	6 (2–17)	3 (2–6)	** < 0.001**
Sex (male)	42,589 (80.5%)	514 (68.2%)	42,075 (80.7%)	** < 0.001**
Obesity	6,360 (12.0%)	53 (7.0%)	6,307 (12.1%)	** < 0.001**
*Comorbidities*				
Cancer	542 (1.0%)	27 (3.6%)	515 (1.0%)	** < 0.001**
Heart failure	17,597 (33.3%)	438 (58.1%)	17,159 (32.9%)	** < 0.001**
Atrial fibrillation /flutter	7,739 (14.6%)	225 (29.8%)	7,512 (14.4%)	** < 0.001**
COPD	3,575 (6.8%)	80 (10.6%)	3,495 (6.7%)	** < 0.001**
Essential arterial hypertension	33,174 (62.7%)	285 (37.8%)	32,889 (63.1%)	** < 0.001**
Chronic renal insufficiency	6,835 (12.9%)	210 (27.9%)	6,625 (12.7%)	** < 0.001**
Diabetes mellitus	15,555 (29.4%)	274 (36.3%)	15,1281 (29.3%)	**0.001**
PAD	4,012 (7.6%)	103 (13.7%)	3,909 (7.5%)	** < 0.001**
Charlson index	3 (2–4)	6 (5–8)	3 (2–5)	** < 0.001**
*CTO PCI approaches*				
Contralateral	32,010 (60.5%)	491 (65.1%)	31,519 (98.5%)	** < 0.001**
Contralateral double	15,811 (29.9%)	223 (29.6%)	15,588 (29.9%)	**0.871**
Retrograd	10,291 (19.5%)	102 (13.5%)	10,189 (19.5%)	** < 0.001**
RetroEx	1,444 (2.7%)	21 (2.8%)	1,423 (2.7%)	**0.924**
*Serious adverse events during hospitalization*				
Ischaemic stroke	299 (0.6%)	29 (3.8%)	270 (0.5%)	** < 0.001**
Transfusion of erythrocytes	2,238 (4.2%)	311 (41.2%)	1,927 (3.7%)	** < 0.001**
Pneumonia	1,580 (3.0%)	196 (26.0%)	1,384 (2.7%)	** < 0.001**
Pericardial effusion	851 (1.6%)	47 (6.2%)	804 (1.5%)	** < 0.001**
Hemopericardium	288 (0.4%)	17 (7.5%)	211 (0.4%)	** < 0.001**
Acute renal failure	1,727 (3.3%)	250 (33.2%)	1,477 (2.8%)	** < 0.001**
Ventricular arrythmia	1,077 (2.0%)	80 (10.6%)	997 (1.9%)	** < 0.001**
Deep vein thrombosis	214 (0.4%)	10 (1.3%)	204 (0.4%)	**0.001**
Pulmonary embolism	89 (0.2%)	7 (0.9%)	82 (0.2%)	** < 0.001**
Myocardial infarction	8,571 (16.2%)	536 (71.1%)	8,035 (15.4%)	** < 0.001**
Shock	1,364 (2.6%)	483 (64.1%)	881 (1.7%)	** < 0.001**
Cardio-pulmonary resuscitation	1,090 (2.1%)	453 (60.1%)	637 (1.2%)	** < 0.001**
COVID-19	8 (0.1%)	0	8 (0.1%)	**1.000**

*COPD*  chronic obstructive pulmonary disease

Values in bold indicate that the difference is statistically significance (*p* < 0.05)

### Predictors of in-hospital fatality

Overall, 754 (1.4%) patients with CTO PCI died during the in-hospital stay and in-hospital case-fatality grew exponentially with age (β 0.82 [95% CI 0.73–0.90]; *p* < 0.001). A multivariate logistic regression model identified several independent predictors of in-hospital case-fatality (Table 2).

**Table 2:** Impact of baseline characteristics, comorbidities, clinical presentation and complications on in-hospital mortality

Parameters	All patients with CTO PCI (*n* = 52,879; 754 patients died in-hospital [1.4%])			
	Univariate		Multivariate (adjusted for age, sex, cancer, heart failure, COPD, arterial hypertension, renal insufficiency and diabetes mellitus)	
	OR (95% CI)	*P*-value	OR (95% CI)	*P*-value
Age	1.1 (1.07–1.09)	**0.001**	1.6 (1.06–1.07)	** < 0.001**
Sex (male)	1.9 (1.67–2.28)	** < 0.001**	1.4 (1.2–1.7)	** < 0.001**
Obesity	0.55 (0.42–0.73)	** < 0.001**	0.56 (0.42–0.74)	** < 0.001**
*Comorbidities*				
Cancer	3.72 (2.51–5.52)	** < 0.001**	2.33 (1.56–3.49)	** < 0.001**
Heart failure	2.82 (2.44–3.27)	** < 0.001**	1.76 (1.51–2.06)	** < 0.001**
Atrial fibrillation /flutter	2.53 (2.16–2.96)	** < 0.001**	1.35 (1.14–1.59)	** < 0.001**
COPD	1.65 (1.31–2.09)	** < 0.001**	1.23 (0.97–1.56)	**0.083**
Essential arterial hypertension	0.36 (0.31–0.41)	** < 0.001**	0.39 (0.34–0.46)	** < 0.001**
Chronic renal insufficiency	2.65 (2.26–3.12)	** < 0.001**	1.29 (1.09–1.59)	**0.003**
Diabetes mellitus	1.38 (1.19–1.60)	**0.001**	1.18 (1.01–1.38)	**0.036**
PAD	1.95 (1.58–2.41)	** < 0.001**	1.52 (1.27–1.89)	** < 0.001**
*CTO PCI approach*				
Contralateral	1.22 (1.05–1.42)	**0.010**	1.19 (1.20–1.38)	**0.027**
Contralateral double	0.98 (0.84–1.15)	**0.844**	0.92 (0.78–1.08)	**0.317**
Retrograd	0.64 (0.52–0.79)	**0.001**	0.76 (0.61–0.94)	**0.010**
RetroEx	1.02 (0.66–1.58)	**0.926**	1.39 (0.89–2.16)	**0.144**
*Serious adverse events through hospitalization*				
Ischaemic stroke	7.68 (5.20–11.35)	** < 0.001**	5.11 (3.40–7.70)	** < 0.001**
Transfusion of erythrocytes	18.28 (15.71–21.29)	** < 0.001**	11.51 (9.77–13.56)	** < 0.001**
Pericardial effusion	4.24 (3.13–5.75)	** < 0.001**	3.05 (3.13–5.75)	** < 0.001**
Haemopericardium	5.67 (3.44–9.35)	** < 0.001**	3.99 (2.37–6.72)	** < 0.001**
Pneumonie	12.88 (10.86–15.28)	** < 0.001**	7.10 (5.89–8.56)	** < 0.001**
Acute renal failure	17.00 (14.49–19.97)	** < 0.001**	10.25 (8.56–12.26)	** < 0.001**
Deep vein thrombosis	3.42 (1.81–6.48)	** < 0.001**	2.34 (1.21–4.52)	**0.011**
Pulmonary embolism	5.95 (2.7–12.91)	** < 0.001**	3.77 (1.69–8.42)	**0.001**
Myocardial infarction	13.49 (11.51–15.82)	** < 0.001**	10.24 (8.70–12.06)	** < 0.001**
Shock	103.67 (88.02–122.02)	** < 0.001**	80.35 (67.42–95.77)	** < 0.001**
Cardio-pulmonary resuscitation	121.65 (103.11–143.52)	** < 0.001**	97.45 (81.67–116.26)	** < 0.001**

*COPD*  Chronic obstructive pulmonary disease, *PAD*  peripheral artery disease, *OR* Odds ratio, *CI* Confidence interval

Values in bold indicate that the difference is statistically significance at least in the multivariate regression model (*p* < 0.05)

Patients’ characteristics associated with increased in-hospital death included age, female sex and comorbidities such as cancer, whereas obesity and arterial hypertension were associated with favourable in-hospital course (Table 2). If CTO recanalization was performed during myocardial infarction, case-fatality was more than tenfold increased (OR 10.24, 95%CI 8.70–12.60, *P* < 0.001). If ischaemic stroke, pericardial effusion, hemopericardium, pneumonia, acute renal failure or pulmonary embolism occurred during the hospitalization, case-fatality was increased substantially. Shock and cardio-pulmonary resuscitation were the strongest predictors for in-hospital mortality (Table 2).

### Regional trends of patients with CTO recanalization

**Fig. 2:**
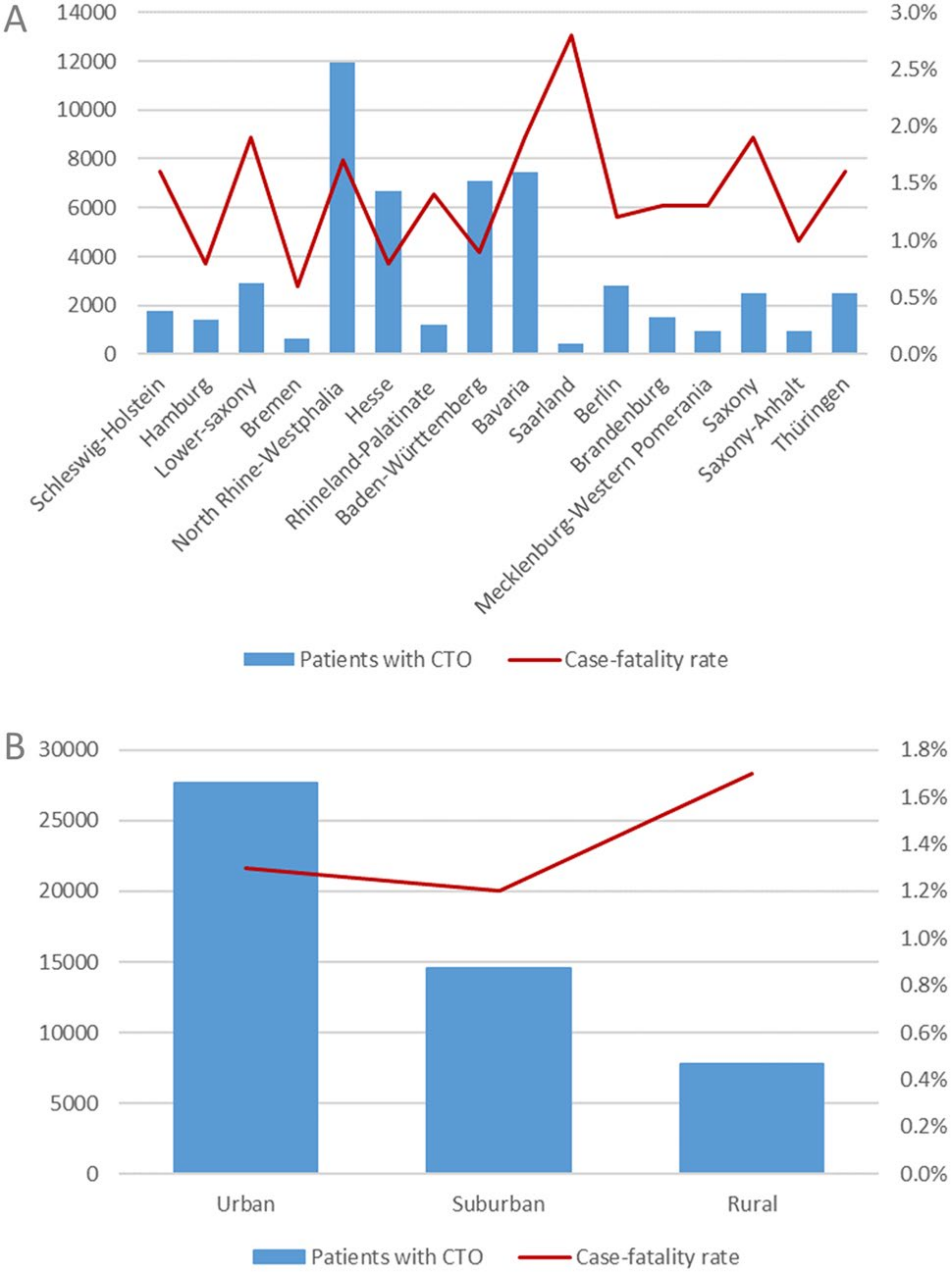
Trends regarding absolute numbers and case-fatality rates and mechanical ventilation stratified for regional (**A**) and urban–rural (**B**) differences in patients with CTO PCI

Most patients with CTO recanalization were treated in hospitals of urban areas (*n* = 27,668) accompanied by a lower case-fatality rate (1.3%) compared to suburban (1.2%) settings and hospitals localized in rural areas (1.7%) (Fig. 2B). Noticeably, we detected regional differences in the federal structure of Germany: The lowest case-fatality was observed in the federal states of Bremen (0.6%) and Hamburg (0.8%) among all federal states in Germany. The highest rate of case fatality was observed in Saarland (2.8%), in which also the lowest total numbers of CTO recanalization procedures were performed (Fig. 2A).

## Discussion

The penetration, trends, associations and immediate clinical implications associated with CTO recanalization are currently debated. Our study evaluates in-hospital data of 52,879 patients undergoing CTO recanalization during the observational period between 2009 and 2020 in the German nationwide inpatient sample to investigate trends of patients’ characteristics, complications and predictors of in-hospital case-fatality.

The main findings of our study are the following: (1) the annual number of CTO procedures increased significantly in our study period from 2009 to 2020 with (2) a significant decrease in case-fatality; (3) age, female sex and comorbidities as cancer were found as strong predictor for an increased in-hospital fatality and (4) case-fatality was lower in urban/suburban areas compared to hospitals in rural regions.

Small study populations reported increased CTO PCI procedures during the last decade, however data from large samples are limited [22, 23]. Our analysis of the German nationwide inpatient cohort showed a progressive increase in the annual number of CTO recanalization from 2009 to 2020. It can be assumed that procedural and technical progress and the recanalization success rates during the last years are driving factors for this increasing penetration. Improvements regarding catheter techniques and material, the use of intravascular imaging and coronary atherectomy with plaque modification such as rotational, orbital or laser atherectomy, shockwave lithoplasty and dedicated treatment algorithms have increased the success rate from 80% in 2008 to currently about 90% [14, 24] despite an increase in the complexity of the CTO-lesions treated. Given the higher complexity, however, the contrast volume, fluoroscopy duration, costs, and procedural duration increased [24]. In analogy with previous publications [23–25], in our German nationwide sample, the in-hospital case-fatality decreased significantly from 2.2% in 2009 to 1.4% in 2020. Several issues regarding timing, indication, and benefit of CTO procedures remain, limiting the proportion of patients undergoing revascularization [4, 15, 23]. Our analysis showed that mainly men (80.5%) and patients older than 50 years underwent CTO PCI. Mortality was affected by age, cancer and other comorbidities such as heart failure, atrial fibrillation, COPD, chronic renal insufficiency, diabetes mellitus and PAD, even after adjustment, this remains associated with a higher in-hospital fatality. The most common in-hospital complications were pneumonia (3%), renal failure (3.3%), and ventricular tachycardia (2%). Besides these, we found more often ischaemic strokes (3.8%), pericardial effusion (6.2%) and hemopericardium (7.5%) among the non-survivors. These rare complications occur in less than 1% of procedures, as previously shown by other groups [5, 26, 27]. In our collective, we found a significant association of CTO PCI with mortality when PCI was performed in patients with myocardial infarction (71.1%), cardiogenic shock (64.1%, or cardiopulmonary resuscitation (60.1%).

Patients with CTO should be treated at specialized centres with experienced operators whenever possible [5]. These results were in line with findings from our analysis showing that CTO PCIs were performed more frequently in urban/suburban regions of Germany and mortality rate was lower as compared to rural regions indicating and underlining a centre volume effect.

### Limitations

Detailed data regarding CTO recanalization experience of the centres and operators, CTO PCI techniques and materials are not available in the data set of the Federal Statistical Office of Germany. In this context, we could report about dual-injection CTO recanalization only, because a code regarding a single-injection approach is not available. In general, our analyses are based on ICD- and OPS-codes of hospitalized patients, which might be prone for under reporting or under coding; finally, follow-up data (e.g., hospital readmission, death, and adverse events following discharge) are not available.

## Conclusion

We observed a considerable increase in CTO procedures with decreased in-hospital fatality during the twelve-year observational period. Important predictors for worse in-hospital outcomes after CTO recanalization were age, female sex and cancer. This identification of predictors is crucial regarding optimal patient selection. In-hospital mortality was highest in patients with myocardial infarction or cardiogenic shock, supporting the importance of a staged procedural intervention.

## Data Availability

The dataset generated and/or analyzed during the current study is available from the corresponding author on reasonable request.

## Abbreviations

CAD: Coronary artery disease
CI: Confidence interval
COPD: Chronic obstructive pulmonary disease
CTO: Chronic total occlusion
DRG: Diagnosis Related Groups
ICD: International Classification of Disease
IQR: Interquartile range
NIS: Nationwide inpatient sample
OPS: Surgery and interventional procedure keys (Operationen- und Prozedurenschlüssel)
OR: Odds ratio
PAD: Peripheral artery disease
PCI: Percutaneous coronary intervention

## References

[1] Di Mario C, Mashayekhi KA, Garbo R, Pyxaras SA, Ciardetti N, Werner GS (2022). Recanalisation of coronary chronic total occlusions. EuroIntervention.

[2] Fefer P, Knudtson ML, Cheema AN, Galbraith PD, Osherov AB, Yalonetsky S (2012). Current perspectives on coronary chronic total occlusions: the Canadian Multicenter Chronic Total Occlusions Registry. J Am Coll Cardiol.

[3] Råmunddal T, Hoebers LP, Henriques JPS, Dworeck C, Angerås O, Odenstedt J (2016). Prognostic impact of chronic total occlusions: a report from SCAAR (Swedish Coronary Angiography and Angioplasty Registry). JACC Cardiovasc Interv.

[4] Strauss BH, Knudtson ML, Cheema AN, Galbraith PD, Elbaz-Greener G, Abuzeid W (2021). Canadian multicenter chronic total occlusion registry: ten-year follow-up results of chronic total occlusion revascularization. Circ Cardiovasc Interv.

[5] Suzuki Y, Tsuchikane E, Katoh O, Muramatsu T, Muto M, Kishi K (2017). Outcomes of percutaneous coronary interventions for chronic total occlusion performed by highly experienced Japanese specialists: The first report from the Japanese CTO-PCI expert registry. JACC Cardiovasc Interv.

[6] Sapontis J, Salisbury AC, Yeh RW, Cohen DJ, Hirai T, Lombardi W (2017). Early procedural and health status outcomes after chronic total occlusion angioplasty: a report from the OPEN-CTO registry (Outcomes, Patient Health Status, and Efficiency in Chronic Total Occlusion Hybrid Procedures). JACC Cardiovasc Interv.

[7] Werner GS, Martin-Yuste V, Hildick-Smith D, Boudou N, Sianos G, Gelev V (2018). A randomized multicentre trial to compare revascularization with optimal medical therapy for the treatment of chronic total coronary occlusions. Eur Heart J.

[8] Obedinskiy AA, Kretov EI, Boukhris M, Kurbatov VP, Osiev AG, Ibn Elhadj Z (2018). The Impactor-CTO trial. JACC Cardiovasc Interv.

[9] Borgia F, Viceconte N, Ali O, Stuart-Buttle C, Saraswathyamma A, Parisi R (2012). Improved cardiac survival, freedom from MACE and angina-related quality of life after successful percutaneous recanalization of coronary artery chronic total occlusions. Int J Cardiol.

[10] Jones DA, Weerackody R, Rathod K, Behar J, Gallagher S, Knight CJ (2012). Successful recanalization of chronic total occlusions is associated with improved long-term survival. JACC Cardiovasc Interv.

[11] Lee S-W, Lee PH, Ahn J-M, Park D-W, Yun S-C, Han S (2019). Randomized trial evaluating percutaneous coronary intervention for the treatment of chronic total occlusion. Circulation.

[12] Henriques JPS, Hoebers LP, Råmunddal T, Laanmets P, Eriksen E, Bax M (2016). Percutaneous intervention for concurrent chronic total occlusions in patients with STEMI: The EXPLORE trial. J Am Coll Cardiol.

[13] Mashayekhi K, Nührenberg TG, Toma A, Gick M, Ferenc M, Hochholzer W (2018). A randomized trial to assess regional left ventricular function after stent implantation in chronic total occlusion: The REVASC trial. JACC Cardiovasc Interv.

[14] Galassi AR, Werner GS, Boukhris M, Azzalini L, Mashayekhi K, Carlino M (2019). Percutaneous recanalisation of chronic total occlusions: 2019 consensus document from the EuroCTO Club. EuroIntervention.

[15] Banning AP, Serruys P, de Maria GL, Ryan N, Walsh S, Gonzalo N (2022). Five-year outcomes after state-of-the-art percutaneous coronary revascularization in patients with de novo three-vessel disease: final results of the SYNTAX II study. Eur Heart J.

[16] Nikolakopoulos I, Vemmou E, Karacsonyi J, Alaswad K, Karmpaliotis D, Abi Rafeh N (2022). Percutaneous coronary intervention of chronic total occlusions involving a bifurcation: Insights from the PROGRESS-CTO registry. Hellenic J Cardiol.

[17] Azzalini L, Dautov R, Ojeda S, Benincasa S, Bellini B, Giannini F (2017). Procedural and long-term outcomes of percutaneous coronary intervention for in-stent chronic total occlusion. JACC Cardiovasc Interv.

[18] Hernandez-Suarez DF, Azzalini L, Moroni F, Tinoco de Paula JE, Lamelas P, Campos CM et al (2022) Outcomes of chronic total occlusion percutaneous coronary intervention in patients with prior coronary artery bypass graft surgery: Insights from the LATAM CTO registry. Catheter Cardiovasc Interv 99(2):245–253 10.1002/ccd.30041 PMC8885848 34931448

[19] Khariton Y, Airhart S, Salisbury AC, Spertus JA, Gosch KL, Grantham JA (2018). Health status benefits of successful chronic total occlusion revascularization across the spectrum of left ventricular function: insights from the OPEN-CTO registry. JACC Cardiovasc Interv.

[20] Patterson C, Sapontis J, Nicholson WJ, Lombardi W, Karmpaliotis D, Moses J (2021). Impact of body mass index on outcome and health status after chronic total occlusion percutaneous coronary intervention: Insights from the OPEN-CTO study. Catheter Cardiovasc Interv.

[21] Salisbury AC, Sapontis J, Grantham JA, Qintar M, Gosch KL, Lombardi W (2017). Outcomes of chronic total occlusion percutaneous coronary intervention in patients with diabetes: insights from the open CTO registry. JACC Cardiovasc Interv.

[22] Othman H, Seth M, Zein R, Rosman H, Lalonde T, Yamasaki H (2020). Percutaneous coronary intervention for chronic total occlusion-The Michigan experience: insights from the BMC2 registry. JACC Cardiovasc Interv.

[23] Brilakis ES, Banerjee S, Karmpaliotis D, Lombardi WL, Tsai TT, Shunk KA (2015). Procedural outcomes of chronic total occlusion percutaneous coronary intervention: a report from the NCDR (National Cardiovascular Data Registry). JACC Cardiovasc Interv.

[24] Konstantinidis NV, Werner GS, Deftereos S, Di Mario C, Galassi AR, Buettner JH (2018). Temporal trends in chronic total occlusion interventions in Europe. Circ Cardiovasc Interv.

[25] Kostantinis S, Simsek B, Karacsonyi J, Alaswad K, Krestyaninov O, Khelimskii D (2022). In-hospital outcomes and temporal trends of percutaneous coronary interventions for chronic total occlusion. EuroIntervention.

[26] Moroni F, Brilakis ES, Azzalini L (2021). Chronic total occlusion percutaneous coronary intervention: managing perforation complications. Expert Rev Cardiovasc Ther.

[27] Xenogiannis I, Gkargkoulas F, Karmpaliotis D, Alaswad K, Jaffer FA, Yeh RW (2020). Temporal trends in chronic total occlusion percutaneous coronary interventions: insights From the PROGRESS-CTO registry. J Invasive Cardiol.

